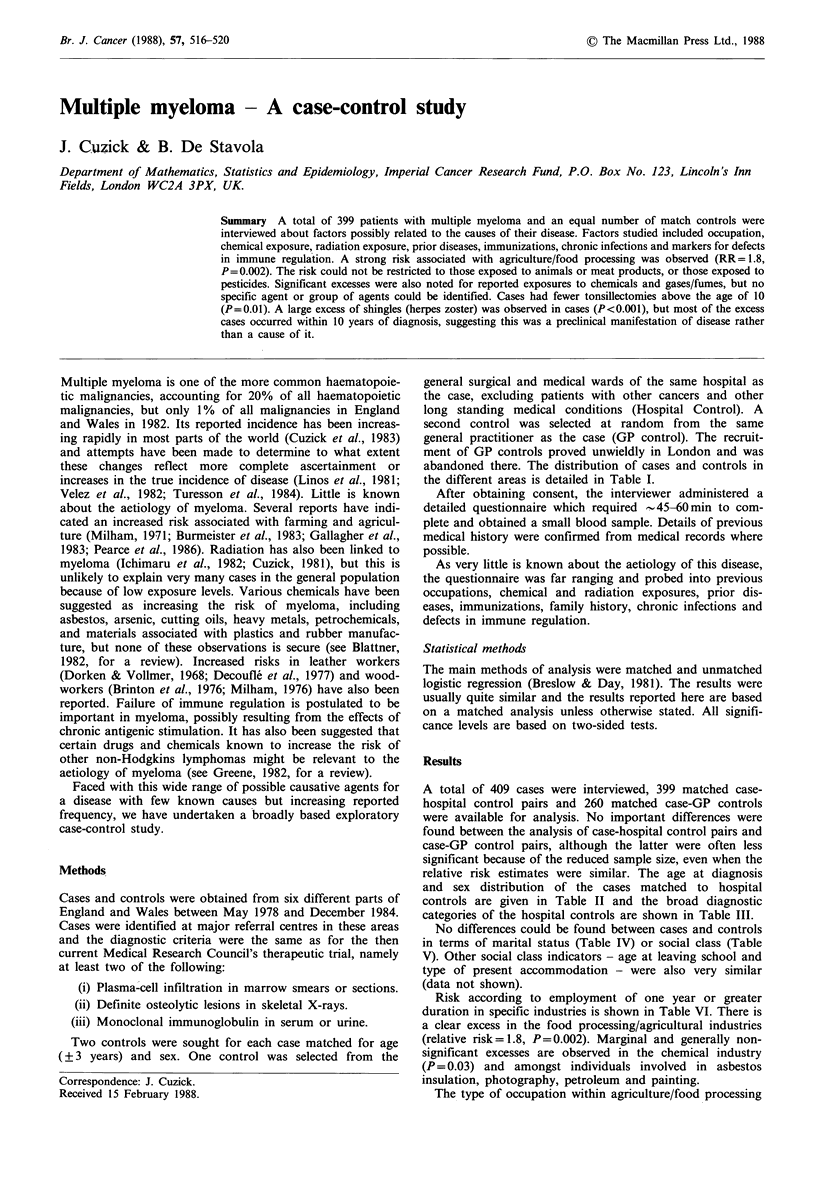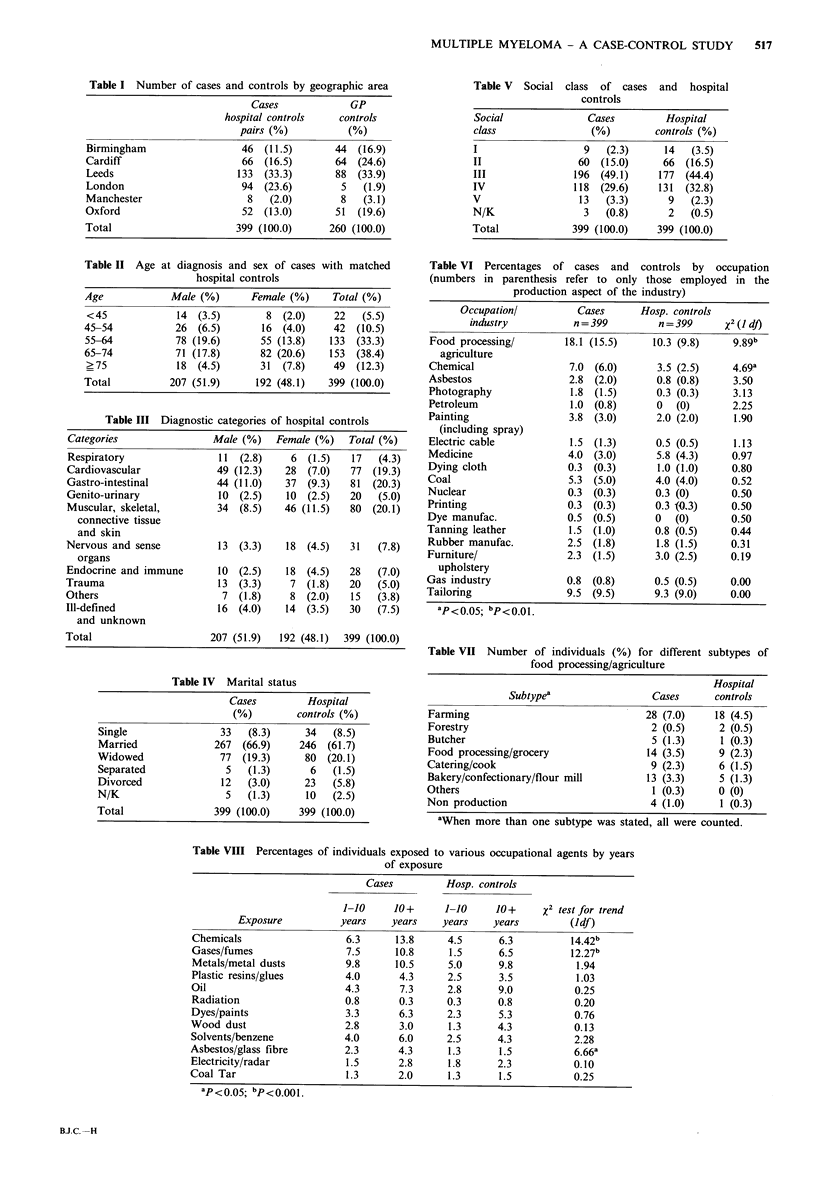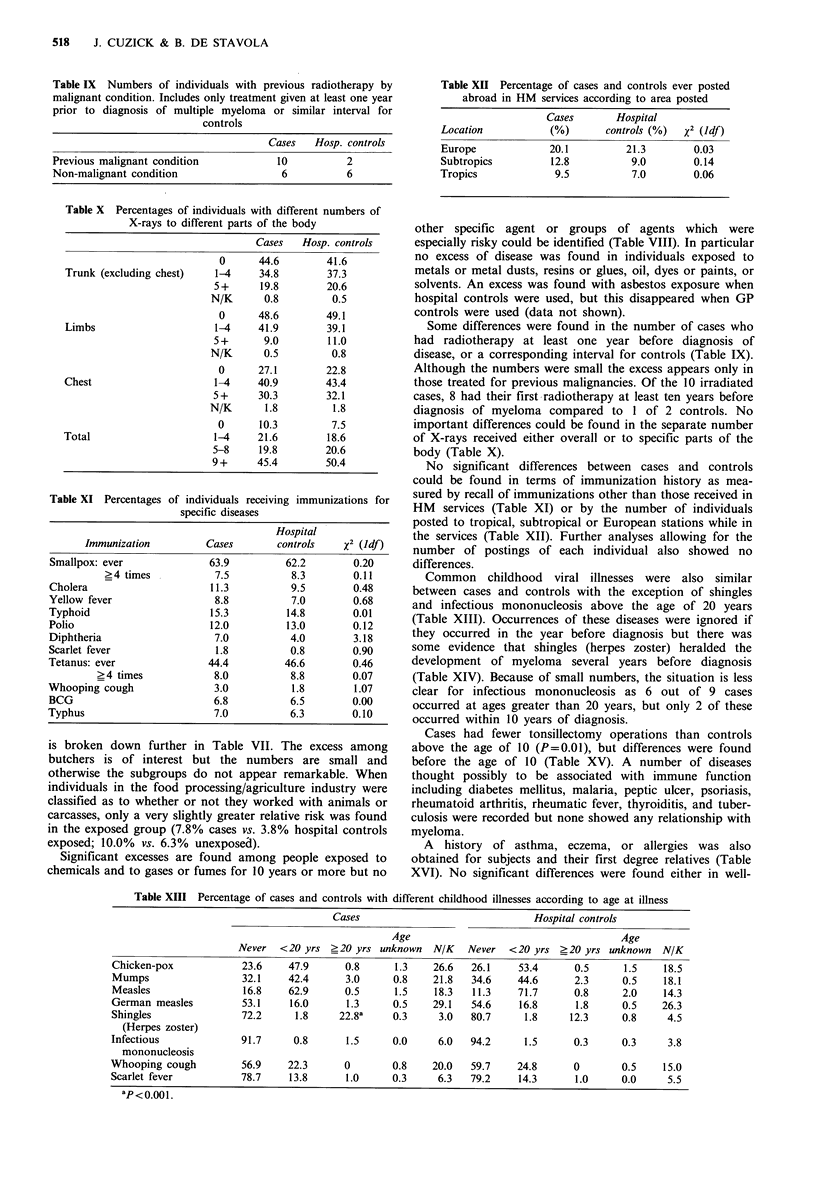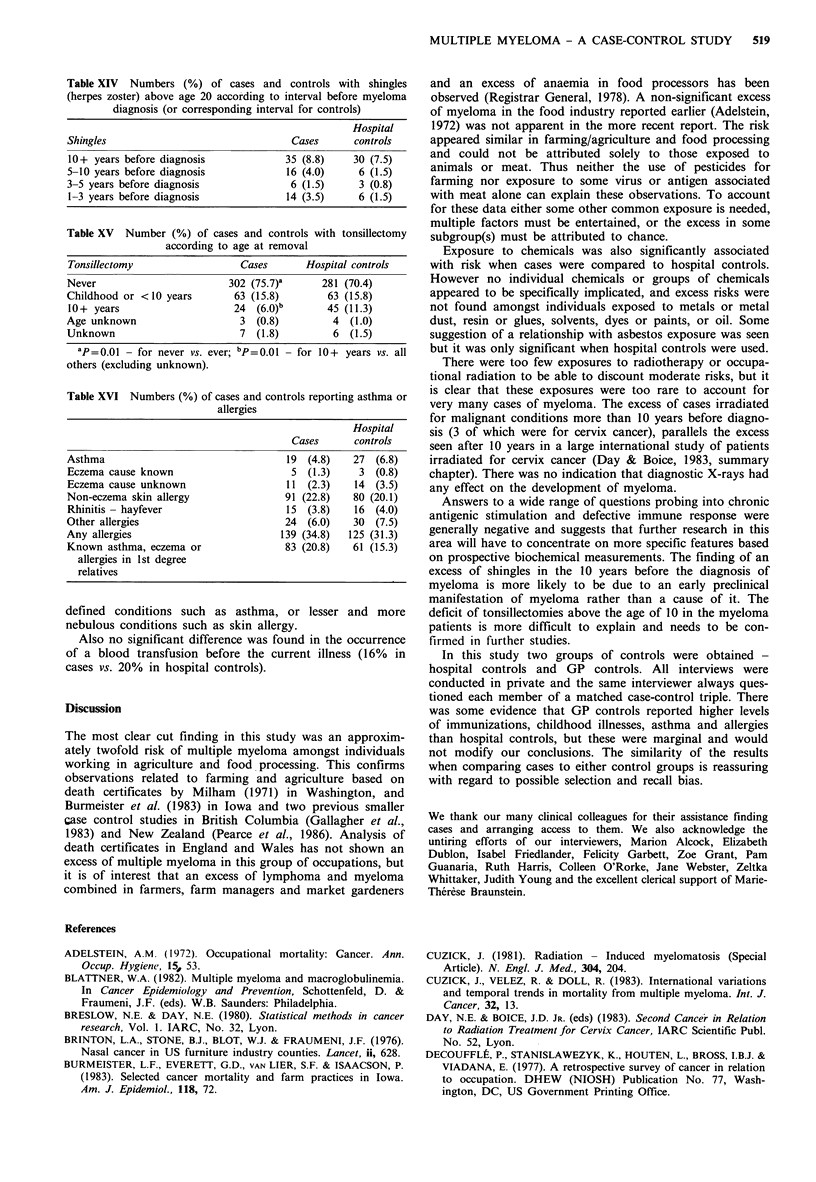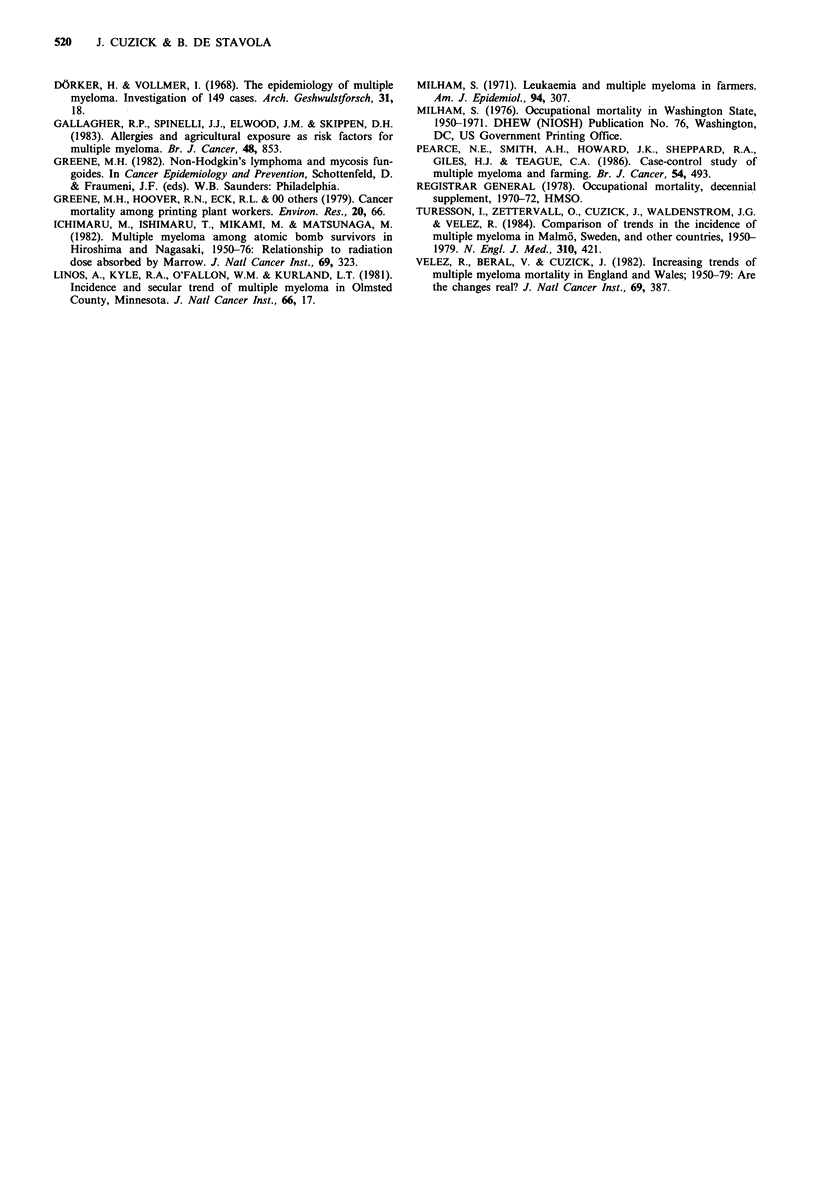# Multiple myeloma--a case-control study.

**DOI:** 10.1038/bjc.1988.118

**Published:** 1988-05

**Authors:** J. Cuzick, B. De Stavola

**Affiliations:** Department of Mathematics, Statistics and Epidemiology, Imperial Cancer Research Fund, London, UK.

## Abstract

A total of 399 patients with multiple myeloma and an equal number of match controls were interviewed about factors possibly related to the causes of their disease. Factors studied included occupation, chemical exposure, radiation exposure, prior diseases, immunizations, chronic infections and markers for defects in immune regulation. A strong risk associated with agriculture/food processing was observed (RR = 1.8, P = 0.002). The risk could not be restricted to those exposed to animals or meat products, or those exposed to pesticides. Significant excesses were also noted for reported exposures to chemicals and gases/fumes, but no specific agent or group of agents could be identified. Cases had fewer tonsillectomies above the age of 10 (P = 0.01). A large excess of shingles (herpes zoster) was observed in cases (P less than 0.001), but most of the excess cases occurred within 10 years of diagnosis, suggesting this was a preclinical manifestation of disease rather than a cause of it.


Br. J. Cancer (1988), 57, 516–520 © The Macmillan Press Ltd., 1988

### Multiple myeloma – A case-control study

J. Cuzick & B. De Stavola

*Department of Mathematics, Statistics and Epidemiology, Imperial Cancer Research Fund, P.O. Box No. 123, Lincoln's Inn Fields, London WC2A 3PX, UK.*

**Summary** A total of 399 patients with multiple myeloma and an equal number of match controls were interviewed about factors possibly related to the causes of their disease. Factors studied included occupation, chemical exposure, radiation exposure, prior diseases, immunizations, chronic infections and markers for defects in immune regulation. A strong risk associated with agriculture/food processing was observed (RR = 1.8, $P = 0.002$). The risk could not be restricted to those exposed to animals or meat products, or those exposed to pesticides. Significant excesses were also noted for reported exposures to chemicals and gases/fumes, but no specific agent or group of agents could be identified. Cases had fewer tonsillectomies above the age of 10 ($P = 0.01$). A large excess of shingles (herpes zoster) was observed in cases ($P < 0.001$), but most of the excess cases occurred within 10 years of diagnosis, suggesting this was a preclinical manifestation of disease rather than a cause of it.

Multiple myeloma is one of the more common haematopoietic malignancies, accounting for 20% of all haematopoietic malignancies, but only 1% of all malignancies in England and Wales in 1982. Its reported incidence has been increasing rapidly in most parts of the world (Cuzick *et al.*, 1983) and attempts have been made to determine to what extent these changes reflect more complete ascertainment or increases in the true incidence of disease (Linos *et al.*, 1981; Velez *et al.*, 1982; Turesson *et al.*, 1984). Little is known about the aetiology of myeloma. Several reports have indicated an increased risk associated with farming and agriculture (Milham, 1971; Burmeister *et al.*, 1983; Gallagher *et al.*, 1983; Pearce *et al.*, 1986). Radiation has also been linked to myeloma (Ichimaru *et al.*, 1982; Cuzick, 1981), but this is unlikely to explain very many cases in the general population because of low exposure levels. Various chemicals have been suggested as increasing the risk of myeloma, including asbestos, arsenic, cutting oils, heavy metals, petrochemicals, and materials associated with plastics and rubber manufacture, but none of these observations is secure (see Blattner, 1982, for a review). Increased risks in leather workers (Dorken & Vollmer, 1968; Decouflé *et al.*, 1977) and woodworkers (Brinton *et al.*, 1976; Milham, 1976) have also been reported. Failure of immune regulation is postulated to be important in myeloma, possibly resulting from the effects of chronic antigenic stimulation. It has also been suggested that certain drugs and chemicals known to increase the risk of other non-Hodgkins lymphomas might be relevant to the aetiology of myeloma (see Greene, 1982, for a review).

Faced with this wide range of possible causative agents for a disease with few known causes but increasing reported frequency, we have undertaken a broadly based exploratory case-control study.

### Methods

Cases and controls were obtained from six different parts of England and Wales between May 1978 and December 1984. Cases were identified at major referral centres in these areas and the diagnostic criteria were the same as for the then current Medical Research Council's therapeutic trial, namely at least two of the following:

(i) Plasma-cell infiltration in marrow smears or sections.
(ii) Definite osteolytic lesions in skeletal X-rays.
(iii) Monoclonal immunoglobulin in serum or urine.

Two controls were sought for each case matched for age (±3 years) and sex. One control was selected from the general surgical and medical wards of the same hospital as the case, excluding patients with other cancers and other long standing medical conditions (Hospital Control). A second control was selected at random from the same general practitioner as the case (GP control). The recruitment of GP controls proved unwieldly in London and was abandoned there. The distribution of cases and controls in the different areas is detailed in Table I.

After obtaining consent, the interviewer administered a detailed questionnaire which required ~45–60 min to complete and obtained a small blood sample. Details of previous medical history were confirmed from medical records where possible.

As very little is known about the aetiology of this disease, the questionnaire was far ranging and probed into previous occupations, chemical and radiation exposures, prior diseases, immunizations, family history, chronic infections and defects in immune regulation.

### Statistical methods

The main methods of analysis were matched and unmatched logistic regression (Breslow & Day, 1981). The results were usually quite similar and the results reported here are based on a matched analysis unless otherwise stated. All significance levels are based on two-sided tests.

### Results

A total of 409 cases were interviewed, 399 matched case-hospital control pairs and 260 matched case-GP controls were available for analysis. No important differences were found between the analysis of case-hospital control pairs and case-GP control pairs, although the latter were often less significant because of the reduced sample size, even when the relative risk estimates were similar. The age at diagnosis and sex distribution of the cases matched to hospital controls are given in Table II and the broad diagnostic categories of the hospital controls are shown in Table III.

No differences could be found between cases and controls in terms of marital status (Table IV) or social class (Table V). Other social class indicators – age at leaving school and type of present accommodation – were also very similar (data not shown).

Risk according to employment of one year or greater duration in specific industries is shown in Table VI. There is a clear excess in the food processing/agricultural industries (relative risk = 1.8, $P = 0.002$). Marginal and generally non-significant excesses are observed in the chemical industry ($P = 0.03$) and amongst individuals involved in asbestos insulation, photography, petroleum and painting.

The type of occupation within agriculture/food processing

Correspondence: J. Cuzick.
Received 15 February 1988.

**Table I** Number of cases and controls by geographic area

| | Cases hospital controls pairs (%) | GP controls (%) |
|---|---|---|
| Birmingham | 46 (11.5) | 44 (16.9) |
| Cardiff | 66 (16.5) | 64 (24.6) |
| Leeds | 133 (33.3) | 88 (33.9) |
| London | 94 (23.6) | 5 (1.9) |
| Manchester | 8 (2.0) | 8 (3.1) |
| Oxford | 52 (13.0) | 51 (19.6) |
| Total | 399 (100.0) | 260 (100.0) |

**Table II** Age at diagnosis and sex of cases with matched hospital controls

| Age | Male (%) | Female (%) | Total (%) |
|---|---|---|---|
| <45 | 14 (3.5) | 8 (2.0) | 22 (5.5) |
| 45–54 | 26 (6.5) | 16 (4.0) | 42 (10.5) |
| 55–64 | 78 (19.6) | 55 (13.8) | 133 (33.3) |
| 65–74 | 71 (17.8) | 82 (20.6) | 153 (38.4) |
| ≧75 | 18 (4.5) | 31 (7.8) | 49 (12.3) |
| Total | 207 (51.9) | 192 (48.1) | 399 (100.0) |

**Table III** Diagnostic categories of hospital controls

| Categories | Male (%) | Female (%) | Total (%) |
|---|---|---|---|
| Respiratory | 11 (2.8) | 6 (1.5) | 17 (4.3) |
| Cardiovascular | 49 (12.3) | 28 (7.0) | 77 (19.3) |
| Gastro-intestinal | 44 (11.0) | 37 (9.3) | 81 (20.3) |
| Genito-urinary | 10 (2.5) | 10 (2.5) | 20 (5.0) |
| Muscular, skeletal, connective tissue and skin | 34 (8.5) | 46 (11.5) | 80 (20.1) |
| Nervous and sense organs | 13 (3.3) | 18 (4.5) | 31 (7.8) |
| Endocrine and immune | 10 (2.5) | 18 (4.5) | 28 (7.0) |
| Trauma | 13 (3.3) | 7 (1.8) | 20 (5.0) |
| Others | 7 (1.8) | 8 (2.0) | 15 (3.8) |
| Ill-defined and unknown | 16 (4.0) | 14 (3.5) | 30 (7.5) |
| Total | 207 (51.9) | 192 (48.1) | 399 (100.0) |

**Table IV** Marital status

| | Cases (%) | Hospital controls (%) |
|---|---|---|
| Single | 33 (8.3) | 34 (8.5) |
| Married | 267 (66.9) | 246 (61.7) |
| Widowed | 77 (19.3) | 80 (20.1) |
| Separated | 5 (1.3) | 6 (1.5) |
| Divorced | 12 (3.0) | 23 (5.8) |
| N/K | 5 (1.3) | 10 (2.5) |
| Total | 399 (100.0) | 399 (100.0) |

**Table V** Social class of cases and hospital controls

| Social class | Cases (%) | Hospital controls (%) |
|---|---|---|
| I | 9 (2.3) | 14 (3.5) |
| II | 60 (15.0) | 66 (16.5) |
| III | 196 (49.1) | 177 (44.4) |
| IV | 118 (29.6) | 131 (32.8) |
| V | 13 (3.3) | 9 (2.3) |
| N/K | 3 (0.8) | 2 (0.5) |
| Total | 399 (100.0) | 399 (100.0) |

**Table VI** Percentages of cases and controls by occupation (numbers in parenthesis refer to only those employed in the production aspect of the industry)

| Occupation/industry | Cases n=399 | Hosp. controls n=399 | $\chi^2$ (1 df) |
|---|---|---|---|
| Food processing/agriculture | 18.1 (15.5) | 10.3 (9.8) | 9.89[b] |
| Chemical | 7.0 (6.0) | 3.5 (2.5) | 4.69[a] |
| Asbestos | 2.8 (2.0) | 0.8 (0.8) | 3.50 |
| Photography | 1.8 (1.5) | 0.3 (0.3) | 3.13 |
| Petroleum | 1.0 (0.8) | 0 (0) | 2.25 |
| Painting (including spray) | 3.8 (3.0) | 2.0 (2.0) | 1.90 |
| Electric cable | 1.5 (1.3) | 0.5 (0.5) | 1.13 |
| Medicine | 4.0 (3.0) | 5.8 (4.3) | 0.97 |
| Dying cloth | 0.3 (0.3) | 1.0 (1.0) | 0.80 |
| Coal | 5.3 (5.0) | 4.0 (4.0) | 0.52 |
| Nuclear | 0.3 (0.3) | 0.3 (0) | 0.50 |
| Printing | 0.3 (0.3) | 0.3 (0.3) | 0.50 |
| Dye manufac. | 0.5 (0.5) | 0 (0) | 0.50 |
| Tanning leather | 1.5 (1.0) | 0.8 (0.5) | 0.44 |
| Rubber manufac. | 2.5 (1.8) | 1.8 (1.5) | 0.31 |
| Furniture/upholstery | 2.3 (1.5) | 3.0 (2.5) | 0.19 |
| Gas industry | 0.8 (0.8) | 0.5 (0.5) | 0.00 |
| Tailoring | 9.5 (9.5) | 9.3 (9.0) | 0.00 |

[a]$P<0.05$; [b]$P<0.01$.

**Table VII** Number of individuals (%) for different subtypes of food processing/agriculture

| Subtype[a] | Cases | Hospital controls |
|---|---|---|
| Farming | 28 (7.0) | 18 (4.5) |
| Forestry | 2 (0.5) | 2 (0.5) |
| Butcher | 5 (1.3) | 1 (0.3) |
| Food processing/grocery | 14 (3.5) | 9 (2.3) |
| Catering/cook | 9 (2.3) | 6 (1.5) |
| Bakery/confectionary/flour mill | 13 (3.3) | 5 (1.3) |
| Others | 1 (0.3) | 0 (0) |
| Non production | 4 (1.0) | 1 (0.3) |

[a]When more than one subtype was stated, all were counted.

**Table VIII** Percentages of individuals exposed to various occupational agents by years of exposure

| | Cases | | Hosp. controls | | |
|---|---|---|---|---|---|
| Exposure | 1–10 years | 10+ years | 1–10 years | 10+ years | $\chi^2$ test for trend (1df) |
| Chemicals | 6.3 | 13.8 | 4.5 | 6.3 | 14.42[b] |
| Gases/fumes | 7.5 | 10.8 | 1.5 | 6.5 | 12.27[b] |
| Metals/metal dusts | 9.8 | 10.5 | 5.0 | 9.8 | 1.94 |
| Plastic resins/glues | 4.0 | 4.3 | 2.5 | 3.5 | 1.03 |
| Oil | 4.3 | 7.3 | 2.8 | 9.0 | 0.25 |
| Radiation | 0.8 | 0.3 | 0.3 | 0.8 | 0.20 |
| Dyes/paints | 3.3 | 6.3 | 2.3 | 5.3 | 0.76 |
| Wood dust | 2.8 | 3.0 | 1.3 | 4.3 | 0.13 |
| Solvents/benzene | 4.0 | 6.0 | 2.5 | 4.3 | 2.28 |
| Asbestos/glass fibre | 2.3 | 4.3 | 1.3 | 1.5 | 6.66[a] |
| Electricity/radar | 1.5 | 2.8 | 1.8 | 2.3 | 0.10 |
| Coal Tar | 1.3 | 2.0 | 1.3 | 1.5 | 0.25 |

[a]$P<0.05$; [b]$P<0.001$.

**Table IX** Numbers of individuals with previous radiotherapy by malignant condition. Includes only treatment given at least one year prior to diagnosis of multiple myeloma or similar interval for controls

| | *Cases* | *Hosp. controls* |
|---|---|---|
| Previous malignant condition | 10 | 2 |
| Non-malignant condition | 6 | 6 |

**Table X** Percentages of individuals with different numbers of X-rays to different parts of the body

| | | *Cases* | *Hosp. controls* |
|---|---|---|---|
| | 0 | 44.6 | 41.6 |
| Trunk (excluding chest) | 1–4 | 34.8 | 37.3 |
| | 5+ | 19.8 | 20.6 |
| | N/K | 0.8 | 0.5 |
| | 0 | 48.6 | 49.1 |
| Limbs | 1–4 | 41.9 | 39.1 |
| | 5+ | 9.0 | 11.0 |
| | N/K | 0.5 | 0.8 |
| | 0 | 27.1 | 22.8 |
| Chest | 1–4 | 40.9 | 43.4 |
| | 5+ | 30.3 | 32.1 |
| | N/K | 1.8 | 1.8 |
| | 0 | 10.3 | 7.5 |
| Total | 1–4 | 21.6 | 18.6 |
| | 5–8 | 19.8 | 20.6 |
| | 9+ | 45.4 | 50.4 |

**Table XI** Percentages of individuals receiving immunizations for specific diseases

| *Immunization* | *Cases* | *Hospital controls* | $\chi^2$ *(1df)* |
|---|---|---|---|
| Smallpox: ever | 63.9 | 62.2 | 0.20 |
| ≧4 times | 7.5 | 8.3 | 0.11 |
| Cholera | 11.3 | 9.5 | 0.48 |
| Yellow fever | 8.8 | 7.0 | 0.68 |
| Typhoid | 15.3 | 14.8 | 0.01 |
| Polio | 12.0 | 13.0 | 0.12 |
| Diphtheria | 7.0 | 4.0 | 3.18 |
| Scarlet fever | 1.8 | 0.8 | 0.90 |
| Tetanus: ever | 44.4 | 46.6 | 0.46 |
| ≧4 times | 8.0 | 8.8 | 0.07 |
| Whooping cough | 3.0 | 1.8 | 1.07 |
| BCG | 6.8 | 6.5 | 0.00 |
| Typhus | 7.0 | 6.3 | 0.10 |

is broken down further in Table VII. The excess among butchers is of interest but the numbers are small and otherwise the subgroups do not appear remarkable. When individuals in the food processing/agriculture industry were classified as to whether or not they worked with animals or carcasses, only a very slightly greater relative risk was found in the exposed group (7.8% cases *vs.* 3.8% hospital controls exposed; 10.0% *vs.* 6.3% unexposed).

Significant excesses are found among people exposed to chemicals and to gases or fumes for 10 years or more but no

**Table XII** Percentage of cases and controls ever posted abroad in HM services according to area posted

| *Location* | *Cases* (%) | *Hospital controls* (%) | $\chi^2$ *(1df)* |
|---|---|---|---|
| Europe | 20.1 | 21.3 | 0.03 |
| Subtropics | 12.8 | 9.0 | 0.14 |
| Tropics | 9.5 | 7.0 | 0.06 |

other specific agent or groups of agents which were especially risky could be identified (Table VIII). In particular no excess of disease was found in individuals exposed to metals or metal dusts, resins or glues, oil, dyes or paints, or solvents. An excess was found with asbestos exposure when hospital controls were used, but this disappeared when GP controls were used (data not shown).

Some differences were found in the number of cases who had radiotherapy at least one year before diagnosis of disease, or a corresponding interval for controls (Table IX). Although the numbers were small the excess appears only in those treated for previous malignancies. Of the 10 irradiated cases, 8 had their first radiotherapy at least ten years before diagnosis of myeloma compared to 1 of 2 controls. No important differences could be found in the separate number of X-rays received either overall or to specific parts of the body (Table X).

No significant differences between cases and controls could be found in terms of immunization history as measured by recall of immunizations other than those received in HM services (Table XI) or by the number of individuals posted to tropical, subtropical or European stations while in the services (Table XII). Further analyses allowing for the number of postings of each individual also showed no differences.

Common childhood viral illnesses were also similar between cases and controls with the exception of shingles and infectious mononucleosis above the age of 20 years (Table XIII). Occurrences of these diseases were ignored if they occurred in the year before diagnosis but there was some evidence that shingles (herpes zoster) heralded the development of myeloma several years before diagnosis (Table XIV). Because of small numbers, the situation is less clear for infectious mononucleosis as 6 out of 9 cases occurred at ages greater than 20 years, but only 2 of these occurred within 10 years of diagnosis.

Cases had fewer tonsillectomy operations than controls above the age of 10 ($P=0.01$), but differences were found before the age of 10 (Table XV). A number of diseases thought possibly to be associated with immune function including diabetes mellitus, malaria, peptic ulcer, psoriasis, rheumatoid arthritis, rheumatic fever, thyroiditis, and tuberculosis were recorded but none showed any relationship with myeloma.

A history of asthma, eczema, or allergies was also obtained for subjects and their first degree relatives (Table XVI). No significant differences were found either in well-

**Table XIII** Percentage of cases and controls with different childhood illnesses according to age at illness

| | *Cases* | | | | | *Hospital controls* | | | | |
|---|---|---|---|---|---|---|---|---|---|---|
| | *Never* | *<20 yrs* | *≧20 yrs* | *Age unknown* | *N/K* | *Never* | *<20 yrs* | *≧20 yrs* | *Age unknown* | *N/K* |
| Chicken-pox | 23.6 | 47.9 | 0.8 | 1.3 | 26.6 | 26.1 | 53.4 | 0.5 | 1.5 | 18.5 |
| Mumps | 32.1 | 42.4 | 3.0 | 0.8 | 21.8 | 34.6 | 44.6 | 2.3 | 0.5 | 18.1 |
| Measles | 16.8 | 62.9 | 0.5 | 1.5 | 18.3 | 11.3 | 71.7 | 0.8 | 2.0 | 14.3 |
| German measles | 53.1 | 16.0 | 1.3 | 0.5 | 29.1 | 54.6 | 16.8 | 1.8 | 0.5 | 26.3 |
| Shingles (Herpes zoster) | 72.2 | 1.8 | 22.8[a] | 0.3 | 3.0 | 80.7 | 1.8 | 12.3 | 0.8 | 4.5 |
| Infectious mononucleosis | 91.7 | 0.8 | 1.5 | 0.0 | 6.0 | 94.2 | 1.5 | 0.3 | 0.3 | 3.8 |
| Whooping cough | 56.9 | 22.3 | 0 | 0.8 | 20.0 | 59.7 | 24.8 | 0 | 0.5 | 15.0 |
| Scarlet fever | 78.7 | 13.8 | 1.0 | 0.3 | 6.3 | 79.2 | 14.3 | 1.0 | 0.0 | 5.5 |

[a]$P<0.001$.

**Table XIV** Numbers (%) of cases and controls with shingles (herpes zoster) above age 20 according to interval before myeloma diagnosis (or corresponding interval for controls)

| *Shingles* | *Cases* | *Hospital controls* |
|---|---|---|
| 10+ years before diagnosis | 35 (8.8) | 30 (7.5) |
| 5–10 years before diagnosis | 16 (4.0) | 6 (1.5) |
| 3–5 years before diagnosis | 6 (1.5) | 3 (0.8) |
| 1–3 years before diagnosis | 14 (3.5) | 6 (1.5) |

**Table XV** Number (%) of cases and controls with tonsillectomy according to age at removal

| *Tonsillectomy* | *Cases* | *Hospital controls* |
|---|---|---|
| Never | 302 (75.7)[a] | 281 (70.4) |
| Childhood or <10 years | 63 (15.8) | 63 (15.8) |
| 10+ years | 24 (6.0)[b] | 45 (11.3) |
| Age unknown | 3 (0.8) | 4 (1.0) |
| Unknown | 7 (1.8) | 6 (1.5) |

[a]$P=0.01$ – for never *vs.* ever; [b]$P=0.01$ – for 10+ years *vs.* all others (excluding unknown).

**Table XVI** Numbers (%) of cases and controls reporting asthma or allergies

| | *Cases* | *Hospital controls* |
|---|---|---|
| Asthma | 19 (4.8) | 27 (6.8) |
| Eczema cause known | 5 (1.3) | 3 (0.8) |
| Eczema cause unknown | 11 (2.3) | 14 (3.5) |
| Non-eczema skin allergy | 91 (22.8) | 80 (20.1) |
| Rhinitis – hayfever | 15 (3.8) | 16 (4.0) |
| Other allergies | 24 (6.0) | 30 (7.5) |
| Any allergies | 139 (34.8) | 125 (31.3) |
| Known asthma, eczema or allergies in 1st degree relatives | 83 (20.8) | 61 (15.3) |

defined conditions such as asthma, or lesser and more nebulous conditions such as skin allergy.

Also no significant difference was found in the occurrence of a blood transfusion before the current illness (16% in cases *vs.* 20% in hospital controls).

### Discussion

The most clear cut finding in this study was an approximately twofold risk of multiple myeloma amongst individuals working in agriculture and food processing. This confirms observations related to farming and agriculture based on death certificates by Milham (1971) in Washington, and Burmeister *et al.* (1983) in Iowa and two previous smaller case control studies in British Columbia (Gallagher *et al.*, 1983) and New Zealand (Pearce *et al.*, 1986). Analysis of death certificates in England and Wales has not shown an excess of multiple myeloma in this group of occupations, but it is of interest that an excess of lymphoma and myeloma combined in farmers, farm managers and market gardeners and an excess of anaemia in food processors has been observed (Registrar General, 1978). A non-significant excess of myeloma in the food industry reported earlier (Adelstein, 1972) was not apparent in the more recent report. The risk appeared similar in farming/agriculture and food processing and could not be attributed solely to those exposed to animals or meat. Thus neither the use of pesticides for farming nor exposure to some virus or antigen associated with meat alone can explain these observations. To account for these data either some other common exposure is needed, multiple factors must be entertained, or the excess in some subgroup(s) must be attributed to chance.

Exposure to chemicals was also significantly associated with risk when cases were compared to hospital controls. However no individual chemicals or groups of chemicals appeared to be specifically implicated, and excess risks were not found amongst individuals exposed to metals or metal dust, resin or glues, solvents, dyes or paints, or oil. Some suggestion of a relationship with asbestos exposure was seen but it was only significant when hospital controls were used.

There were too few exposures to radiotherapy or occupational radiation to be able to discount moderate risks, but it is clear that these exposures were too rare to account for very many cases of myeloma. The excess of cases irradiated for malignant conditions more than 10 years before diagnosis (3 of which were for cervix cancer), parallels the excess seen after 10 years in a large international study of patients irradiated for cervix cancer (Day & Boice, 1983, summary chapter). There was no indication that diagnostic X-rays had any effect on the development of myeloma.

Answers to a wide range of questions probing into chronic antigenic stimulation and defective immune response were generally negative and suggests that further research in this area will have to concentrate on more specific features based on prospective biochemical measurements. The finding of an excess of shingles in the 10 years before the diagnosis of myeloma is more likely to be due to an early preclinical manifestation of myeloma rather than a cause of it. The deficit of tonsillectomies above the age of 10 in the myeloma patients is more difficult to explain and needs to be confirmed in further studies.

In this study two groups of controls were obtained – hospital controls and GP controls. All interviews were conducted in private and the same interviewer always questioned each member of a matched case-control triple. There was some evidence that GP controls reported higher levels of immunizations, childhood illnesses, asthma and allergies than hospital controls, but these were marginal and would not modify our conclusions. The similarity of the results when comparing cases to either control groups is reassuring with regard to possible selection and recall bias.

We thank our many clinical colleagues for their assistance finding cases and arranging access to them. We also acknowledge the untiring efforts of our interviewers, Marion Alcock, Elizabeth Dublon, Isabel Friedlander, Felicity Garbett, Zoe Grant, Pam Guanaria, Ruth Harris, Colleen O'Rorke, Jane Webster, Zeltka Whittaker, Judith Young and the excellent clerical support of Marie-Thérèse Braunstein.

### References

ADELSTEIN, A.M. (1972). Occupational mortality: Cancer. *Ann. Occup. Hygiene*, **15**, 53.

BLATTNER, W.A. (1982). Multiple myeloma and macroglobulinemia. In *Cancer Epidemiology and Prevention*, Schottenfeld, D. & Fraumeni, J.F. (eds). W.B. Saunders: Philadelphia.

BRESLOW, N.E. & DAY, N.E. (1980). *Statistical methods in cancer research*, Vol. 1. IARC, No. 32, Lyon.

BRINTON, L.A., STONE, B.J., BLOT, W.J. & FRAUMENI, J.F. (1976). Nasal cancer in US furniture industry counties. *Lancet*, **ii**, 628.

BURMEISTER, L.F., EVERETT, G.D., VAN LIER, S.F. & ISAACSON, P. (1983). Selected cancer mortality and farm practices in Iowa. *Am. J. Epidemiol.*, **118**, 72.

CUZICK, J. (1981). Radiation – Induced myelomatosis (Special Article). *N. Engl. J. Med.*, **304**, 204.

CUZICK, J., VELEZ, R. & DOLL, R. (1983). International variations and temporal trends in mortality from multiple myeloma. *Int. J. Cancer*, **32**, 13.

DAY, N.E. & BOICE, J.D. JR. (eds) (1983). *Second Cancer in Relation to Radiation Treatment for Cervix Cancer*, IARC Scientific Publ. No. 52, Lyon.

DECOUFFLÉ, P., STANISLAWEZYK, K., HOUTEN, L., BROSS, I.B.J. & VIADANA, E. (1977). A retrospective survey of cancer in relation to occupation. DHEW (NIOSH) Publication No. 77, Washington, DC, US Government Printing Office.

DÖRKER, H. & VOLLMER, I. (1968). The epidemiology of multiple myeloma. Investigation of 149 cases. *Arch. Geshwulstforsch*, **31**, 18.

GALLAGHER, R.P., SPINELLI, J.J., ELWOOD, J.M. & SKIPPEN, D.H. (1983). Allergies and agricultural exposure as risk factors for multiple myeloma. *Br. J. Cancer*, **48**, 853.

GREENE, M.H. (1982). Non-Hodgkin's lymphoma and mycosis fungoides. In *Cancer Epidemiology and Prevention*, Schottenfeld, D. & Fraumeni, J.F. (eds). W.B. Saunders: Philadelphia.

GREENE, M.H., HOOVER, R.N., ECK, R.L. & 00 others (1979). Cancer mortality among printing plant workers. *Environ. Res.*, **20**, 66.

ICHIMARU, M., ISHIMARU, T., MIKAMI, M. & MATSUNAGA, M. (1982). Multiple myeloma among atomic bomb survivors in Hiroshima and Nagasaki, 1950–76: Relationship to radiation dose absorbed by Marrow. *J. Natl Cancer Inst.*, **69**, 323.

LINOS, A., KYLE, R.A., O'FALLON, W.M. & KURLAND, L.T. (1981). Incidence and secular trend of multiple myeloma in Olmsted County, Minnesota. *J. Natl Cancer Inst.*, **66**, 17.

MILHAM, S. (1971). Leukaemia and multiple myeloma in farmers. *Am. J. Epidemiol.*, **94**, 307.

MILHAM, S. (1976). Occupational mortality in Washington State, 1950–1971. DHEW (NIOSH) Publication No. 76, Washington, DC, US Government Printing Office.

PEARCE, N.E., SMITH, A.H., HOWARD, J.K., SHEPPARD, R.A., GILES, H.J. & TEAGUE, C.A. (1986). Case-control study of multiple myeloma and farming. *Br. J. Cancer*, **54**, 493.

REGISTRAR GENERAL (1978). Occupational mortality, decennial supplement, 1970–72, HMSO.

TURESSON, I., ZETTERVALL, O., CUZICK, J., WALDENSTROM, J.G. & VELEZ, R. (1984). Comparison of trends in the incidence of multiple myeloma in Malmö, Sweden, and other countries, 1950–1979. *N. Engl. J. Med.*, **310**, 421.

VELEZ, R., BERAL, V. & CUZICK, J. (1982). Increasing trends of multiple myeloma mortality in England and Wales; 1950–79: Are the changes real? *J. Natl Cancer Inst.*, **69**, 387.